# The effect of surface treatment and thermocycling on the shear bond strength of orthodontic brackets to the Y-TZP zirconia ceramics: A systematic review

**DOI:** 10.1590/2177-6709.26.5.e212118.oar

**Published:** 2021-10-25

**Authors:** Tamzid AHMED, Nashid FAREEN, Mohammad Khursheed ALAM

**Affiliations:** 1 Bangladesh Dental College, Department of Science of Dental Materials (Dhaka, Bangladesh).; 2 Bangladesh Dental College, Department of Conservative Dentistry and Endodontics (Dhaka, Bangladesh).; 3 Jouf University, College of Dentistry, Department of Orthodontics (Sakaka, Kingdom of Saudi Arabia).

**Keywords:** Y-TZP zirconia, Orthodontic brackets, Shear bond strength, Surface treatment

## Abstract

**Introduction:**

Various surface pre-treatment methods have been adapted to optimize the bonding between the zirconia ceramics and the orthodontic brackets.

**Objective:**

This review is aimed at systematically analyzing the relevant data available in the literature, to find out the most effective and durable bonding protocol.

**Methods:**

Database search was conducted in PubMed, Scopus, and ScienceDirect, during September 2020. The review was conducted according to the PRISMA guidelines.

**Results:**

Based on the inclusion criteria, 19 articles were selected for qualitative analysis. Meta-analysis could not be performed due to the heterogeneity of the methodology among the studies. Most of the studies scored medium risk of bias. Compared to the untreated surface, surface pretreatments like sandblasting and lasers were advantageous. Primers and universal adhesive were mostly used as an adjunct to the mechanical pretreatment of the zirconia surface. In most studies, thermocycling seemed to lower the shear bond strength (SBS) of the orthodontic brackets.

**Conclusion:**

Based on this qualitative review, surface pretreatments with lasers and sandblasting can be suggested to optimize the bracket bond strength. To clarify this finding, meta-analysis is anticipated. Hence, high heterogeneity of the included studies demands standardization of the methodology.

## RESUMO

**Introdução:**

Diferentes métodos de pré-tratamento da superfície foram adaptados para otimizar a colagem entre os braquetes ortodônticos e as cerâmicas de zircônia.

**Objetivo:**

A presente revisão teve como objetivo analisar, de forma sistemática, os dados relevantes na literatura, para buscar o protocolo de colagem mais efetivo e duradouro.

**Métodos:**

As buscas foram feitas em setembro de 2020, nas seguintes bases de dados: PubMed, Scopus e ScienceDirect. A revisão foi feita de acordo com as diretrizes do PRISMA.

**Resultados:**

Com base nos critérios de inclusão, 19 artigos foram selecionados para análise qualitativa. A metanálise não pôde ser feita, devido à heterogeneidade na metodologia dos estudos incluídos. A maior parte dos estudos apresentou risco de viés moderado. Comparadas às superfícies não tratadas, as superfícies com pré-tratamento usando jateamento ou *laser* apresentaram vantagem. *Primers* e adesivos universais foram mais usados como adjuntos ao pré-tratamento mecânico da superfície de zircônia. Na maioria dos estudos, a termociclagem parece ter reduzido a resistência ao cisalhamento dos braquetes ortodônticos.

**Conclusões:**

Com base nessa revisão qualitativa, os pré-tratamentos de superfície com *laser* e jateamento podem ser sugeridos para otimizar a resistência ao cisalhamento dos braquetes. Para esclarecer esse achado, uma metanálise é necessária. Para isso, faz-se necessária a padronização da metodologia, para lidar com a alta heterogeneidade dos estudos incluídos.

## INTRODUCTION

With the increasing demand for esthetics and the introduction of the CAD/CAM devices, zirconia ceramics have gained much popularity in modern Dentistry. Zirconia claimed superiority in terms of mechanical properties, biocompatibility, precision and esthetics.^1^ ^,^ ^2^ The yttrium-stabilized tetragonal zirconia polycrystal (Y-TZP) is the form most commonly used in Dentistry. The material is known for the phase transformation from tetragonal to monoclinic due to stressful conditions, achieving fracture resistance.^3^ It has been used extensively to make inlays, onlays, crowns, post and core systems, and frameworks for porcelain fused to zirconia restorations.^4^ Hence, with the increasing use of zirconia restorative materials, it is becoming more common for orthodontists to bond orthodontic brackets to a Y-TZP surface. However, due to the chemical inertness and resistance to acid-etching, bonding composite resin to the zirconia surface can be challenging. Thus, various surface treatment methods -such as mechanical, chemical, or combined - have been introduced to improve the bond strength of zirconia ceramics. 

The *in-vitro* shear bond strength (SBS) is the most commonly used test, due to its simplicity and resemblance to the shear force exerted during mastication. In addition, aiming at testing the durability of these methods, the material can be exposed to water at a certain temperature, simulating an oral environment for a determined period - a procedure known as thermocycling. 

Previously, reviews were mostly based on the bond strength between the luting cement and the zirconium surface; overlooking the impact of orthodontic brackets.^5^ ^-^ ^7^ Therefore, this study is aimed to perform a systematic review of the *in-vitro* studies that reported the effect of different surface conditioning and aging on the shear bond strength of orthodontic brackets to the Y-TZP zirconia ceramics.

## MATERIAL AND METHODS

### SEARCH STRATEGY

The review was conducted by following the Preferred Reporting Items for Systematic Reviews and Meta-Analyses (PRISMA) guidelines. The research question of this review was: *Which surface treatment is best suited to optimize the shear bond strength of orthodontic brackets to Y-TZP zirconia ceramics?* The question was developed concerning the following PICO (population, intervention, comparison, and outcome): Y-TZP zirconia, surface treatment, thermocycling, no thermocycling, comparison between surface treatment methods with the resultant shear bond strength (SBS), respectively. 

The electronic databases PubMed, Scopus, and ScienceDirect were searched, using the following keywords: orthodontic brackets, shear bond strength, zirconium, ceramic surface, and thermocycling. Additionally, Google Scholar was searched with the same keywords. The references of the selected studies were also checked for any missing relevant studies. All searches were performed in September 2020. The search was confined to the original articles written in English language, published between the years 2001 to 2020. All the duplicates were resolved by the Endnote X7 software (Thompson Reuters, Philadelphia, PA, USA). The search strategy is summarized in Table 1. 

**Table 1:** Database search strategy.

Name of Database	Last date of Search	Keywords	Studies found	Studies selected
Pubmed Central	12/09/2020	Orthodontic brackets, shear bond strength, zirconium, ceramic surface, thermocycling	28	12
Scopus	12/09/2020		08	08
ScienceDirect	12/09/2020		15	2
Google Scholar	15/09/2020		1680	86

### STUDY SELECTION

Two independent reviewers screened the titles and abstracts of all the studies found. The articles were selected according to the following inclusion and exclusion criteria:

### INCLUSION CRITERIA

1) Studies that bonded orthodontic brackets to Y-TZP surface.
2) Studies that conditioned the surface before bonding.
3) Studies with a well-designed shear bond strength test.
4)*In-vitro* experimental and observational studies.
5) Full-text original articles.

### EXCLUSION CRITERIA

1)*In-vivo* studies and clinical trials.
2) Studies testing materials other than orthodontic brackets and Y-TZP zirconia, such as resins or composite cement, buccal tubes, enamel, dentine, zirconium fused to porcelain or lithium silicate infused with zirconium, etc.

The agreement between both reviewers was confirmed by Cohen’s kappa statistics.^8^ A third reviewer was consulted in the event of any disagreement. 

### DATA EXTRACTION

Two reviewers independently reviewed the full-text articles and extracted the following data in tabulated form: study year, sample size, surface treatment method, adhesives and brackets used, storage, method of thermocycling, and the resultant shear bond strength (Table 2). Agreement between the reviewers was assessed by Cohen’s Kappa statistics. The third reviewer’s opinion was considered to resolve any disagreement. 

**Table 2:** Data extraction.

Study	Sample	Surface treatment	Adhesive	Brackets	Storage	Thermocycling	Bond strength (MPa)
Akay et al.^12^ (2020)	48	G1) Er:YAG laser (2W) G2) Nd:YAG laser (2W) G3) Sandblasting (SiO_2_) G4) 9.6% hydrofluoric acid	Transbond XT light cured composite	Maxillary central incisor metal brackets	37ºC deionized water for 30 days	2,000 cycles between 5±2 - 55±2ºC with 30s dwelling time	G1: 5.5 ± 0.79 G2: 4.88 ± 0.82 G3: 7.42 ± 0.92 G4: 3.58 ± 0.75
Ju et al.^4^ (2020)	80	G1) Sandblasting G2) Sandblasting + Clearfil ceramic primer on zirconia surface G3) Sandblasting + Clearfil ceramic primer on bracket base G4) Sandblasting + Clearfil ceramic primer on both zirconia surface and bracket base	Transbond XT primer + Transbond XT light cure composite	Monocrystalline ceramic brackets	At 37ºC and humidity 100% for 24h.	10,000 cycles between 5º - 55ºC with 30s dwelling time	Mean and standard deviation not included
Ju et al.^2^ (2019)	60	G1) Sandblasting + Clearfil Ceramic primer + orthodontic primer G2) Sandblasting + universal adhesive G3) Sandblasting + Clearfil ceramic primer + universal adhesive	Transbond XT light cured composite	Maxillary central incisor ceramic brackets	37ºC and relative humidity 100% incubator for 24h.	10,000 cycles between 5º - 55ºC with 30s dwelling time	Before thermocycling: G1: 9.78 ± 1.94 G2: 9.86 ± 1.33 G3: 9.16 ± 0.78 After thermocycling: G1: 8.16 ± 1.78 G2: 4.99 ± 0.99 G3: 4.31 ± 1.02
Mehmeti et al.^26^ (2019)	48	G1) 37% phosphoric acid for 120s + silane primer G2) 5% hydrofluoric acid + silane primer	Transbond XT primer + Transbond XT light cure composite	i) Metal brackets ii) Polycrystalline ceramic brackets	Not mentioned	5,800 cycles between 5º - 55ºC with 10s dwelling time	Metal brackets: G1: 10.85 ± 5.84 G2: 8.52 ± 4.72 Ceramic brackets: G1: 11.84 ± 7.30 G2: 8.99 ± 5.36
Cetik et al.^15^ (2019)	40	G1) Sandblasting + Silane primer G2) Er:YAG laser + Silane primer	Brack Fix primer + Brack 1Fix light cured composite	i) Mandibular anterior metal brackets ii) Mandibular anterior ceramic brackets	Not done	Not done	Metal brackets: G1: 23.29 ± 5.34 G2: 21.59 ± 4.03 Ceramic brackets: G1: 20.06 ± 4.05 G2: 17.55 ± 3.88
Douara et al.^27^ (2019)	45	G1) Sandblasting (Al_2_O_3_) + universal bonding resin (Assure plus) G2) Sandblasting (Al_2_O_3_) + Silane + Universal bonding resin (Assure plus) + Silane G3) Sandblasting (Al_2_O_3_) + 4% hydrofluoric acid + Silane	Transbond XT primer+ Transbond XT light cure composite resin	Monocrystalline ceramic brackets	37ºC distilled water for 24 hrs.	500 cycles between 5º - 55ºC with 30s dwelling time	G1: 2.50 ± 0.75 G2: 7.81 ± 2.81 G3: 8.15 ± 2.41
Garcia-Sanz et al.^9^ (2018)	90	G1) No treatment G2) Sandblasting G3) Femtosecond Ti:Sapphire laser output power- 300 mW, inter-groove distance 60 µm G4) Femtosecond Ti:Sapphire laser output power- 200 mW, inter-groove distance 100 µm G5) Femtosecond Ti:Sapphire laser output power- 40 mW, inter-groove distance 60 µm. G6) Femtosecond Ti:Sapphire laser output power- 200 mW, inter-groove distance 60 µm	Transbond XT primer + Transbond XT light cure composite	Maxillary incisor metal brackets	37ºC distilled water for 24 hrs.	Not done	G1: 3.87 ± 0.77 G2: 4.25 ± 0.51 G3: 5.92 ± 1.12 G4: 3.74 ± 0.10 G5: 3.91 ± 0.53 G6: 5.68 ± 0.94
Byeon et al.^14^ (2017)	130	G1) Polishing G2) Sandblasting G3) Sandblasting + Silane primer G4) Sandblasting + MDP primer G5) Sandblasting + MDP containing silane primer	Transbond XT light cured composite	Maxillary central incisor metal brackets	37 ± 1ºC distilled water for 24 hrs.	5,000 cycles between 5º - 55ºC with 30s dwelling time	Before thermocycling: G1: 2.6 ± 1.1 G2: 4.98 ± 1.28 G3: 5.13 ± 0.85 G4: 11.92 ± 1.51 G5: 13.36 ± 2.31 After thermocycling: G1: 0.70 ± 0.4 G2: 0.8 ± 0.3 G3: 1.5 ± 0.4 G4: 5.4 ± 3.5 G5: 5.7 ± 1.2
Garcia-Sanz et al.^17^ (2017)	300	G1) No treatment G2) Sandblasting G3) Tribochemical silica coating + Silane primer G4) Femtosecond laser (200 mW) G5) Sandblasting + Femtosecond laser (200 mW)	Transbond XT primer + Transbond XT light cure composite	i) Maxillary central incisor metal brackets. ii) Maxillary central incisor ceramic brackets.	Not mentioned	Not mentioned	Metal brackets: G1: 4.23 ± 0.89 G2: 4.46 ± 1.21 G3: 5.99 ± 1.86 G4: 6.72 ± 2.30 G5: 7.22 ± 2.73 Ceramic brackets: G1: 20.06 ± 2.34 G2: 25.01 ± 4.45 G3: 21.62 ± 6.48 G4: 23.18 ± 6.51 G5: 29.22 ± 8.20
Mehmeti et al.^24^ (2017)	20	37% phosphoric acid for 120s	Transbond XT primer + Transbond XT light cure composite	G1: Metal brackets G2: Polycrystalline ceramic brackets	Waterbath for 24 hrs.	Not done	G1: 7.35 ± 3.41 G2: 4.66 ± 1.78
Lee et al.^28^ (2017)	50	G1) Sandblasting G2) Sandblasting + Metal/Zirconia primer G3) Sandblasting + Z-Prime plus G4) Sandblasting + Zirconia liner G5) Sandblasting + Scotchbond universal adhesive	Transbond XT primer + Transbond XT light cured composite	Ceramic brackets	37ºC distilled water for 24 hrs.	2,000 cycles between 5º - 55ºC for 1 minute	G1: 1.07 ± 0.81 G2: 5.16 ± 0.83 G3: 10.47 ± 2.89 G4: 9.55 ± 1.75 G5: 13.85 ± 1.48
Kim et al.^19^ (2017)	124	G1) Sandblasting (Al_2_O_3_) + Silane primer G2) Sandblasting (Al_2_O_3_) + Zirconia prime plus G3) Sandblasting (Al_2_O_3_) + universal bonding resin G4) Sandblasting (SiO_2_) + Silane primer G5) Sandblasting (SiO_2_) + Zirconia prime plus G6) Sandblasting (SiO_2_) + universal bonding resin	Transbond XT primer + Transbond XT light cured composite	Mandibular anterior metal brackets	100% relative humidity for 7 days	2,000 cycles between 5º-55ºC for 1 minute with 20s dwelling time.	Non-thermocycled: G1: 11.4 ± 5.8 G2: 21.6 ± 3.3 G3: 22.9 ± 6.5 G4: 19.7 ± 4.1 G5: 20.5 ± 5.4 G6: 24.2 ± 2.8 Thermocycled: G1: 13.7 ± 5.0 G2: 20 ± 4.9 G3: 22.5 ± 6.9 G4: 25 ± 5 G5: 24.1 ± 3.5 G6: 26.2 ± 3.1
Kim et al.^20^ (2017)	160	G1) Sandblasting + ESPE-sil G2) Sandblasting + Alloy primer G3) Sandblasting + Clearfil ceramic primer G4) Sandblasting + Single bond universal primer G5) Tribochemical silica coating + ESPE-sil G6) Tribochemical silica coating + Alloy primer G7) Tribochemical silica coating + Clearfil ceramic primer G8) Tribochemical silica coating + Single bond universal primer	Transbond XT light cured composite	Maxillary central incisor metal brackets	Deionized water 37º C for 24 h	5000 cycles between 5º - 55ºC with 30s dwelling time	Non-thermocycled: G1: 6.6 ± 2.6 G2: 15.9 ± 5.2 G3: 13.1 ± 3.4 G4: 16.7 ± 5.6 G5: 7.9 ± 3.4 G6: 17 ± 3.9 G7: 15.9 ± 5.7 G8: 19.4 ± 4.5 Thermocycled: G1: 5.2 ± 1.4 G2: 14.3 ± 2.9 G3: 12.9 ± 4.3 G4: 15.2 ± 5.3 G5: 5.8 ± 1.6 G6: 8.6 ± 1.7 G7: 14.8 ± 3.9 G8: 7.1 ± 0.9
Amer and Rayyan^13^ (2018)	60	G1) No surface treatment G2) Sandblasting G3) Soflex disc	i) Clearfil ceramic primer + Panavia F 2.0 adhesive resin cement ii) Rely X U200 self adhesive resin cement	Lower second premolar metal brackets	37ºC distilled water for 24 hrs.	500 cycles between 5º - 55ºC with 30s dwelling time	Clearfil + Panvia F 2.0: G1: 0 G2: 20.8 ± 4.8 G3: 12.3 ± 2.8 Rely X U200: G1: 0 G2: 16.7 ± 4.6 G3: 11.6 ± 3
Ihsan and Al-Dabagh^29^ (2017)	40	G1) Z prime plus primer G2) Sandblasting (Al_2_O_3_) + Z prime plus primer G3) Nd:YAG laser (0.888 W, 5s.) + Z-prime plus primer G4) Nd:YAG laser (0.444 W, 10s) + Z-prime plus primer	Light-cured composite	Central incisor sapphire brackets	37ºC distilled water for 24 hrs.	Not done	G1: 11.08 ± 1.96 G2: 22.29 ± 1.18 G3: 30.25 ± 2.31 G4: 30.67 ± 2.33
Hosseini et al.^18^ (2016)	72	G1) No treatment G2) Er:YAG laser 1.5 W G3) Er:YAG laser 2.5 W G4) Er:YAG laser 3 W G5) Sandblasting G6) Silane primer	Transbond XT light cure composite	Maxillary incisor metal brackets	37ºC distilled water for 24 hrs.	500 cycles between 5º - 55ºC with 30s dwelling time	G1: 0.31 ± 0.23 G2: 0.51 ± 0.14 G3: 1.11 ± 0.40 G4: 3.32 ± 1.52 G5: 9.50 ± 2.92 G6: 3.88 ± 2.20
Lee et al.^22^ (2015)	40	G1) Non-glazed + Sandblasting + Zirconia primer G2) Glazing + Sandblasting + etching + Zirconia primer G3) Glazing + sandblasting + etching + porcelain primer G4) Glazing + sandblasting + etching + zirconia primer + porcelain primer	Transbond XT light cured composite	Mandibular incisor metal brackets	37ºC distilled water	2,000 cycles between 5º - 55ºC with 30s dwelling time	G1: 13.7 ± 1.3 G2: 3.7 ± 0.9 G3: 16 ± 2.6 G4: 14.4 ± 1.7
Kwak et al.^21^ (2016)	70	G1 - unglazed): Silicon carbide paper roughening G2) Diamond bur +Z-prime plus primer G3) Pumice + Monobond-S primer G4) 4% hydrofluoric acid + Monobond-S primer G5) Sandblasting (Al_2_O_3_) + Monobond-S primer G6) Sandblasting (Al_2_O_3_) + Z-prime plus primer G7) Sandblasting (SiO_2_) + Monobond-S primer	Transbond XT light cure composite resin	Mandibular incisor metal brackets	37ºC water for 24 hrs	1,000 cycles between 5º - 55ºC	G1: 13.38 ± 2.57 G2: 15.48 ± 3.15 G3: 14.90 ± 2.75 G4: 15.24 ± 3.36 G5: 15.78 ± 2.39 G6: 4.60 ± 1.08 G7: 14.81 ± 2.91
Yassaei et al.^25^ (2015)	72	G1) 9.6% hydrofluoric acid + silane primer G2) Sandblasting (Al_2_O_3_) + Silane primer G3) Er:YAG laser (1W) + Silane primer G4) Er:YAG laser (2W) + Silane primer	Light-cured composite resin	Metallic maxillary central incisor brackets	37ºC water for 24 hrs	500 cycles between 5º - 55ºC with 30s dwelling time	G1: 5.8 ± 0.78 G2: 7.8 ± 1.02 G3: 6.8 ± 0.92 G4: 6.9 ± 1.13

### RISK OF BIAS

The methodological merits of the selected studies were assessed by both reviewers individually. The assessment tool was adapted from previous *in-vitro* systematic reviews and meta-analyses, judging on the following parameters: sample size calculation, presence of control group, use of materials according to the manufacturer’s instruction, standardized sample preparation, surface treatment and bonding done by the same operator, adequate description of thermocycling and the appropriate statistical analysis (i.e., reporting mean, standard deviation and *p*-values).^9^ ^,^ ^10^ Any of these parameters reported by the articles was ticked with “Y” (yes) for the particular section. In case any parameter was missing, was marked with an “N” (no). Articles reporting only one (1) to three (3) of the items were considered as having a high risk of bias; four (4) to five (5) items, as medium risk of bias; and six (6) to seven (7) items, as low risk of bias.^9^ ^,^ ^10^ Again, the interexaminer agreement was analyzed by Cohen’s kappa statistics, and the third reviewer’s opinion was requested in any event of disagreement. 

## RESULTS

### LITERATURE SEARCH

**Figure 1:**
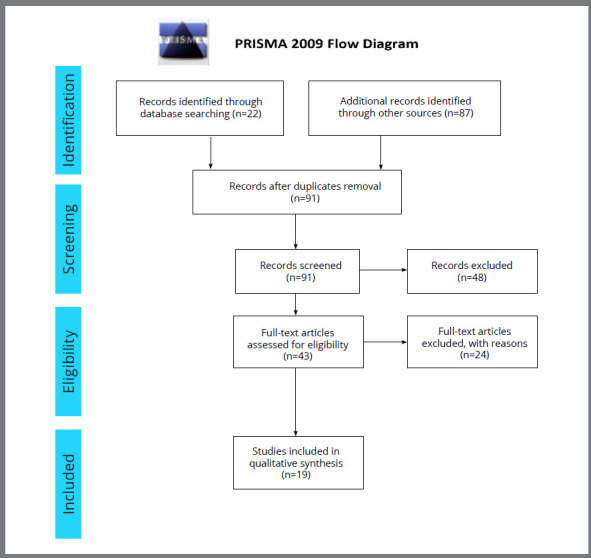
Flow diagram of the study selection strategy.

A total of 109 studies was identified (Table 1). After the removal of the duplicates, 91 articles remained. Upon careful screening of the titles and abstracts, 48 articles were further excluded. The kappa value for interexaminers agreement was *k* = 0.816 (*p*< 0.001). Forty three full-text articles were screened thoroughly for eligibility and 24 articles were excluded for valid reasons (Supplementary Table 1 ). Finally, 19 articles were selected for the review. The PRISMA flow diagram of the study selection procedure is presented in Figure 1. 

**Supplementary table 1:** Reason for exclusion.

Article	Reason for exclusion
Ahluwalia et al., 2013	Zirconium surface was not included
Buyuk et al., 2018	Zirconium surface was not included
Byeon et al., 2016	Full-text article was not found
Di guida et al., 2019	Zirconium surface was not included
Dong et al., 2016	Full-text article was not found
Elsaka et al., 2016	Zirconium surface was not included
Juntavee et al., 2018	Zirconium surface was not included
Kawai et al., 2011	Orthodontic brackets were not included
Kaya et al., 2019	Zirconium surface was not included
Murthy et al., 2014	Orthodontic brackets were not included
Soliman et al., 2018	Zirconium surface was not included
Zhang et al., 2016	Zirconium surface was not included
Pitriani et al., 2019	Zirconium surface not included
Gardiner et al., 2019	Orthodontic buccal tubes included, not brackets
Mehta et al., 2016	Shear bond strength not measured
Mirhashmi et al., 2020	Shear bond strength in MPa units not measured
Abuelenain et al., 2020	Lithium silicate reinforced with zirconia
Ahluwalia et al., 2013	Porcelain fused to zirconia crowns
Amer and Rayyan, 2018	Glazed monolithic zirconia crowns
Bilgic et al., 2013	Porcelain fused to zirconia crowns
Franz et al., 2019	Zirconium oxide sinter ceramics
Ismail and Sheikh, 2018	Porcelain veneered zirconia crown
Oldham et al., 2019	Lithium silicate infused with zirconia
Pouyanfar et al., 2019	Y-TZP zirconia was not included

### QUALITATIVE ASSESSMENT

For the qualitative analysis, the inter-reviewers reliability of the extracted data was confirmed (k = 0.89, *p* < 0.001). Three types of brackets were studied. Metallic brackets were the most common;^11^ ^-^ ^24^ followed by the ceramic^2, 4,15,17,23,24,26,27^, and sapphire brackets.^28^ Both mechanical and chemical methods of surface treatment were identified on the Y-TZP zirconia surface. Mechanical means included lasers, sandblasting, polishing and soflex disc, silicon carbide paper, and diamond bur roughening.^2^ ^,^ ^4^ ^,^ ^12^ ^-^ ^21^ ^,^ ^25^ ^,^ ^26^ ^,^ ^28^ Chemically, acid etching (9.6% hydrofluoric acid, 37% phosphoric acid), application of primers, and universal adhesive were found.^2^ ^,^ ^12^ ^,^ ^14^ ^,^ ^16^ ^-^ ^20^ ^,^ ^22^ ^,^ ^25^ ^,^ ^27^ ^,^ ^28^ Sandblasting was the most found surface treatment. Two types of sandblasting were noticed: conventional (Al_2_O_3_) sandblasting and tribochemical silica (SiO_2_) coated sandblasting.^17^ ^,^ ^19^ ^-^ ^21^ Sandblasting method differed between the studies as the variables like particle size (25µm - 110µm), pressure (0.14 MPa - 0.4 MPa), time (5 seconds - 20seconds), and the distance (10mm - 20mm) were not homogeneous. Three types of lasers were utilized: erbium-doped yttrium aluminum garnet laser (Er:YAG), neodymium-doped yttrium aluminum garnet laser (Nd:YAG), and the femtosecond laser.^12^ ^,^ ^15^ ^-^ ^18^ ^,^ ^25^ ^,^ ^28^ Sandblastings and lasers were also combined with various primers and universal adhesive.^2^ ^,^ ^14^ ^,^ ^15^ ^,^ ^19^ ^,^ ^20^ ^,^ ^22^ ^,^ ^25^ ^,^ ^27^ ^,^ ^28^ Eight studies treated Y-TZP surface with sandblasting only;^4^ ^,^ ^12^ ^,^ ^14^ ^,^ ^16^ ^-^ ^18^ ^,^ ^27^ five studies combined sandblasting with silane primer,^14^ ^,^ ^17^ ^,^ ^19^ ^,^ ^20^ ^,^ ^25^ five studies combined sandblasting with MDP (methacryloyloxydecyl dihydrogen phosphate) primers;^14^ ^,^ ^19^ ^,^ ^20^ ^,^ ^27^ ^,^ ^28^ four studies combined universal adhesive;^2^ ^,^ ^19^ ^,^ ^20^ ^,^ ^27^ three studies combined MDP containing silane primer,^2^ ^,^ ^14^ ^,^ ^20^ and two studies combined zirconia primer.^22^ ^,^ ^27^ Lasers were used alone in four studies;^12^ ^,^ ^16^ ^-^ ^18^ and as an adjunct with MDP primer and silane primer in one study each.^18^ ^,^ ^25^ One study also combined femtosecond laser and sandblasting to treat Y-TZP surface.^17^ Variability was observed in laser settings like power output (40 mW to 3 W); mean energy settings (50mJ to 300mJ), distance (60µm to 10mm), and the application time (5 seconds to 2 minutes). Except for two studies,^18^ ^,^ ^28^ no study was found to treat the Y-TZP surface solely with primers before orthodontic bonding. The trade names and chemical composition of the primers used to treat Y-TZP surface are listed in Table 3. 

**Table 3:** Primers used to treat Y-TZP surface.

Primers	Trade name	Chemical composition
Silane primer	ESPE Sil, Rely X, Reliance porcelain conditioner	3-TMSPMA, Ethanol
MDP Primers	Z-PRIME Plus	MDP, Ethanol
	Zirconia liner	MMA, 10-MDP, 4-methoxyphenol (HQME)
	Primer Alloy	6-(4-vinylbenzyl-n-propyl) amino-1,3,5-triazine-2,4 dithiol (VBATDT), 10-MDP, acetone
MDP containing Silane primer	Clearfil Ceramic Primer	10-MDP, 3-TMSPMA, Ethanol.
Universal adhesive	Clearfil S3 Bond, Scotchbond universal adhesive, Single Bond Universal	10-MDP, bis-GMA, HEMA, hydrophobic demethacrylate, dl-camphorquinone, ethyl alcohol, water, silanated colloidal silica
Orthodontic primer	Transbond XT adhesive primer	TEGMA, bis-GMA, triphenylantimony, 4-(dimethylamino)-benzeneethanol, dl-camphorquinone, ethyl alcohol, water, silanated colloidal silica
Zirconia primer	Metal/Zirconia primer	Tertiary butyl alcohol, methyl isobutyl ketone, phosphoric acid acrylate, benzoylperoxide.

Abbreviations: 3-TMSPMA (3-trimethoxysilylpropyl methacrylate), 10-MDP (10-methacryloyloxydecyl dihydrogen phosphate), Bis-GMA (bisphenol-A-diglycidyl methacrylate), HEMA (hydroxyethyl methacrylate), TEGMA (triethylene glycol dimethacrylate), MMA (methyl methacrylate), HQME (hydroquinone monoethyl ether).

Two studies did not thermocycled their samples.^16^ ^,^ ^17^ Different protocols of thermocycling were observed between the studies; mostly at the range of 500-10,000 cycles, at 5 - 55ºC temperature with dwell time of 20 to 30 seconds. The 37ºC distilled water for 24 hours was the most common method of storage before bonding.^13^ ^,^ ^14^ ^,^ ^16^ ^,^ ^18^ ^,^ ^21^ ^,^ ^22^ ^,^ ^25^ ^-^ ^28^ Transbond XT^©^ primer and light-curing composite were widely used as an adhesive.^2^ ^,^ ^9^ ^,^ ^12^ ^,^ ^14^ ^,^ ^17^ ^-^ ^20^ ^,^ ^22^ ^,^ ^27^

Qualitative analyses of the included studies are detailed in Table 2. 

### RISK OF BIAS

**Figure 2:**
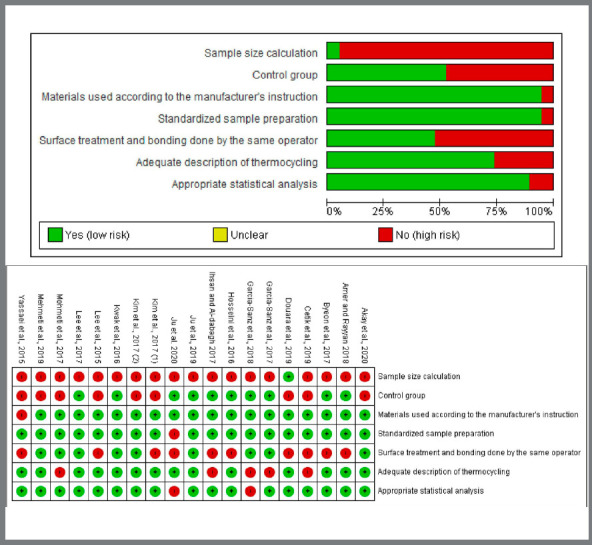
Summary of the risk of bias of the included studies,

Based on the criteria applied for the quality assessment of the selected studies; thirteen (13) studies scored medium risk of bias, three (3) studies scored low risk of bias, and the remaining three (3) studies scored high risk of bias. Sample size calculation was surprisingly absent (except for one selected study). Half of the studies had no control groups and surface treatment. Bonding was done by the same operator in only 47.4% of cases. The frequency of the rest of the parameters was sufficient (Fig 2).

## DISCUSSION

In the examined literature, different surface treatment protocols have been studied extensively regarding the adherence between the orthodontic brackets and the Y-TZP zirconia ceramics. Previously, several reviews on the ceramic surface conditioning and other testing parameters were published either with or without quantitative analysis.^5^ ^-^ ^7^ ^,^ ^9^ The reviews were neither of the specific types of ceramic surface nor on the effect of orthodontic brackets. Regarding mechanical testing protocols, the shear bond strength (SBS) test is the most popular method because it is less complicated and produces results with a smaller standard deviation.^5^ Therefore, the effect of surface treatment methods on the SBS of the orthodontic brackets, particularly on the Y-TZP zirconia ceramic surface, was reviewed. 

In this review, 19 studies were included for in-depth analysis testing SBS of orthodontic brackets to Y-TZP surface bonded with light-cure resin/composites. Meta-analysis could not be performed due to the high heterogeneity of the included studies, limiting the study outcome. This high variability may be due to the variability in sample size, storage, thermocycling, sandblasting, different laser settings, bracket materials (i.e. metal, ceramic), bracket anatomy, and base design. Maxillary central incisor metallic bracket was mostly studied.^12^ ^,^ ^14^ ^,^ ^16^ ^-^ ^18^ ^,^ ^20^ ^,^ ^25^ On reviewing the studies that compared the SBS between the metallic and the ceramic brackets, mixed outcomes were observed.^15^ ^,^ ^17^ ^,^ ^26^ A previously published research found that the ceramic brackets had greater bond strength to the Y-TZP surface than the metal brackets.^17^ On 37% phosphoric acid etching, metallic brackets exhibited significantly better SBS than ceramic brackets.^24^ In a study, 37% phosphoric acid was combined with silane primer to enhance the SBS of the ceramic brackets; but the result was not significant.^26^ In comparison to phosphoric acid etching, the hydrofluoric acid etching did not improve the SBS in both metallic and ceramic brackets.^26^ In fact, surface pretreatment with hydrofluoric acid resulted in lower SBS of orthodontic brackets, in comparison to sandblasting, Er:YAG laser irradiation, and 37% phosphoric acid etching.^12^ ^,^ ^26^ Due to the possibility of weakening the ceramic surface, the use of hydrofluoric acid was condemned.^26^ Both metallic and ceramic brackets exhibited higher SBS to the sandblasted and laser-irradiated surfaces in different studies. One study reported higher SBS of ceramic brackets on sandblasted, tribochemical silica-coated and femtosecond laser irradiated surfaces.^17^ Conversely, another study reported higher SBS of metallic brackets on the sandblasted and the Er:YAG laser-irradiated surface.^15^ Surprisingly, comparative analysis of the SBS of metal and ceramic brackets on Y-TZP surface treated by various primers could not be found. 

Sandblasting was found as the most common surface treatment method among the included studies. It promotes mechanical bonding by creating surface irregularities through air abrasion, using either alumina or silica-coated alumina particles. The latter procedure exhibited greater SBS in many studies.^12^ ^,^ ^19^ ^-^ ^21^ The CoJet™ system that applies silica-coated alumina particles not only abrades the ceramic surface, but also creates silica enriched outer surface favorable for silanization.^6^ Surface irregularities created by both of these particles were similar in previous studies.^17^ ^,^ ^20^ However, the surface free energy was greater in silica-coated sandblasting.^19^ ^,^ ^20^ Therefore, the additional application of primers promoting chemical adhesion might have contributed to the higher SBS. Following both forms of sandblasting, primers like silane, MDP, MDP containing silane primers or single-step universal adhesives were applied to optimize the SBS.^2^ ^,^ ^4^ ^,^ ^19^ ^,^ ^20^ ^,^ ^22^ The primers and universal adhesives that contain MDP are capable of chemically bond to zirconia. The bonding occurs between the phosphate ester of MDP and the hydroxyl groups over the zirconia surface.^14^ The silane primers had lower SBS in comparison to the universal bonding resin, MDP, or MDP containing silane primers, as they do not form a chemical bond with zirconia unless the surface is coated with silica.^14^ ^,^ ^19^ ^,^ ^20^

Mechanical surface treatments such as sandblasting and lasers also have been studied.^12^ ^,^ ^16^ ^-^ ^18^ ^,^ ^25^ ^,^ ^29^ Lasers produce surface roughness by a process called ablation, which involves micro explosions and vaporization.^6^ Both Er:YAG and Nd:YAG laser irradiation of zirconia surface resulted in inferior SBS, compared to sandblasting.^12^ ^,^ ^15^ ^,^ ^18^ ^,^ ^25^ The Er:YAG lasers alone failed to achieve Reynolds’s optimal SBS range of 5.9 - 7.8 MPa.^12^ ^,^ ^18^ ^,^ ^30^ Yet, in combination with silane primer, the SBS was raised to 6.9 MPa.^25^ The additional chemical reaction and wetting ability of the silane may have contributed to this finding. Besides, at high power output (above 200 mJ) the laser generates high heat, which can be detrimental to the surrounding zirconia surface.^31^ The femtosecond lasers, at 200mW power output and 60µm inter-groove distance, generated SBS (5.68 MPa) closer to the optimal level.^15^ In separate studies, the femtosecond laser-treated zirconia surface had better SBS compared to both alumina (Al_2_O_3_) and silica (SiO_2_) coated sandblasting.^16^ ^,^ ^17^ Better SBS was achieved when the femtosecond laser was combined with sandblasting, but not significantly greater than the femtosecond laser alone.^17^ Thereby, additional surface preparation as an adjunct to femtosecond laser can be avoided to save time, cost and patient discomfort. Besides, the laser has no reports of thermal damage due to surface irradiation.^32^

Following thermocycling, there was a noticeable reduction in SBS irrespective of the surface pre-treatment methods.^2^ ^,^ ^14^ ^,^ ^19^ ^,^ ^20^ Biodegradation of the treated Y-TZP surface, bonded brackets, and adhesives may have contributed to this evidence. Hence, the assessment of the bond strength in a simulated clinical environment (i.e., *in-vivo* experiment) is necessary. Thermocycling is an artificial aging procedure to test the long-term effect of bond strength. According to a study, 10,000 cycles of thermocycling are equivalent to one year of usage in the oral cavity.^33^ Application of MDP and MDP containing silane primers resulted in durable and optimal SBS on both forms of sandblasted Y-TZP, even after 10,000 cycles of thermocycling.^2^ ^,^ ^20^ Conversely, in the case of universal adhesives, the SBS was stable up to 2,000 cycles, but degraded significantly after 10,000 cycles.^2^ ^,^ ^20^ ^,^ ^27^ Among lasers, the effect of thermocycling on the Er:YAG and Nd:YAG laser irradiated surfaces were tested. The SBS on Er:YAG laser-treated surface was found to be just clinically acceptable within the range of 500-2,000 cycles.^12^ ^,^ ^25^ In the case of Nd:YAG laser, the SBS was below an acceptable level after thermocycling.^12^ Surprisingly, the effect of thermocycling on the femtosecond laser irradiation could not be found. 

The validation risk of the bias tool utilized in this study could not be confirmed. This fact should be regarded as an important limitation of the study, but the contents of this quality assessment tool seemed to be more justifiable and relevant to the methodology of the selected studies. The absence of meta-analysis is another limitation reflecting the heterogeneity of the studies.

## CONCLUSION

As this review is solely based on the qualitative analysis of the laboratory-based *in-vitro* findings, the results should be interpreted with caution. To answer the research question of this review more precisely, quantitative analysis is deemed necessary. Therefore, standardization of the study protocol is necessary. However, certain points may be advised, in light of this comprehensive review:

a) Hydrofluoric acid etching can be avoided to treat the Y-TZP surface, as it did not remarkably improve the SBS, considering the damage to the ceramic surface.
b) Mechanical pretreatments like sandblasting and lasers are useful, as they both improved the SBS of orthodontic brackets.
c) Tribochemical silica-coated sandblasting with the advantage of chemical adhesion resulted in greater SBS than conventional sandblasting.
d) Among lasers, the femtosecond laser can be suggested as the first choice; although the effect of thermocycling on this laser irradiated zirconia surface is unknown.
e) Use of primers, particularly the MDP and the MDP containing silane primers as an adjunct to the mechanical pretreatments may be justified. There is a concern over the longevity of the universal adhesive.
